# Ozone exposure is associated with acute changes in inflammation, fibrinolysis, and endothelial cell function in coronary artery disease patients

**DOI:** 10.1186/s12940-017-0335-0

**Published:** 2017-11-21

**Authors:** Jaime E. Mirowsky, Martha Sue Carraway, Radhika Dhingra, Haiyan Tong, Lucas Neas, David Diaz-Sanchez, Wayne Cascio, Martin Case, James Crooks, Elizabeth R. Hauser, Z. Elaine Dowdy, William E. Kraus, Robert B. Devlin

**Affiliations:** 1 Department of Chemistry, SUNY College of Environmental Science and Forestry, 1 Forestry Drive, Syracuse, NY 13210 USA; 2 Curriculum in Toxicology, University of North Carolina, Chapel Hill, NC USA; 3 Department of Medicine, Pulmonary and Critical Care Medicine, Durham VA Medical Center, Durham, NC USA; 4 National Health and Environmental Effects Laboratory, US Environmental Protection Agency, Chapel Hill, NC USA; 5 Department of Biomedical Research, National Jewish Health, Denver, CO USA; 6 Division of Biostatistics and Bioinformatics, National Jewish Health, Denver, CO USA; 7 Department of Epidemiology, Colorado School of Public Health, Denver, CO USA; 8 Duke Molecular Physiology Institute, Duke University School of Medicine, Durham, NC USA; 9 Department of Biostatistics and Bioinformatics, Duke University School of Medicine, Durham, NC USA; 10 Cooperative Studies Program Epidemiology Center, Durham Veterans Affairs Medical Center, Durham, NC USA; 11 Division of Cardiology, Department of Medicine, School of Medicine, Duke University, Durham, NC USA

**Keywords:** Ozone, Air pollution, Cardiovascular, Inflammation, Coronary artery disease, Panel study

## Abstract

**Background:**

Air pollution is a major risk factor for cardiovascular disease, of which ozone is a major contributor. Several studies have found associations between ozone and cardiovascular morbidity, but the results have been inconclusive. We investigated associations between ozone and changes across biological pathways associated with cardiovascular disease.

**Methods:**

Using a panel study design, 13 participants with coronary artery disease were assessed for markers of systemic inflammation, heart rate variability and repolarization, lipids, blood pressure, and endothelial function. Daily measurements of ozone and particulate matter (PM_2.5_) were obtained from central monitoring stations. Single (ozone) and two-pollutant (ozone and PM_2.5_) models were used to assess percent changes in measurements per interquartile ranges of pollutants.

**Results:**

Per interquartile increase in ozone, changes in tissue plasminogen factor (6.6%, 95% confidence intervals (CI) = 0.4, 13.2), plasminogen activator inhibitor-1 (40.5%, 95% CI = 8.7, 81.6), neutrophils (8.7% 95% CI = 1.5, 16.4), monocytes (10.2%, 95% CI = 1.0, 20.1), interleukin-6 (15.9%, 95% CI = 3.6, 29.6), large-artery elasticity index (−19.5%, 95% CI = −34.0, −1.7), and the baseline diameter of the brachial artery (−2.5%, 95% CI = −5.0, 0.1) were observed. These associations were robust in the two-pollutant model.

**Conclusions:**

We observed alterations across several pathways associated with cardiovascular disease in 13 coronary artery disease patients following ozone exposures, independent of PM_2.5_. The results support the biological plausibility of ozone-induced cardiovascular effects. The effects were found at concentrations below the EPA National Ambient Air Quality Standards for both ozone and PM_2.5_.

**Electronic supplementary material:**

The online version of this article (10.1186/s12940-017-0335-0) contains supplementary material, which is available to authorized users.

## Background

Air pollution is a major and independent environmental risk factor for cardiovascular disease [1]. Epidemiological studies suggest that the strongest associations are between particulate matter (PM) and cardiovascular morbidity and mortality [2, 3]; however, recent work suggests that ozone also may be associated with negative cardiovascular health effects including coronary death, cardiac arrest, and ischemic stroke [4–6]. To clarify some contrary findings [7, 8] and establish biological plausibility, work is needed to establish potential mechanisms mediating ozone’s adverse cardiovascular health effects [9].

Clinical and toxicological studies have established several possible mechanisms by which PM adversely impacts the cardiovascular system, thus providing biologic plausibility for the epidemiologic studies; similar approaches have begun to be used with ozone using controlled human exposure studies. Under controlled conditions, increases in systemic pro-inflammatory markers were observed in 26 healthy human participants following exposures to ozone and clean air [10, 11]. In addition, researchers have found changes in fibrinolysis markers such as plasminogen activator inhibitor-1, plasminogen, and D-dimer, when young healthy participants were exposed to ozone [11, 12]. Further, researchers have begun looking at changes in cardiovascular biomarkers in larger epidemiological work to support associations between ozone exposure and cardiovascular morbidity and mortality. In 1536 people living in Stockholm, changes in fibrinogen were associated with short-term ambient ozone exposures [13]. Blood pressure, platelet activation markers, and arterial stiffness were also found to be altered with ozone exposures in healthy adults living in China [14]. Additional research of men participating in the Normative Aging Study found changes in heart rate variability parameters with exposures to ambient ozone levels [15].

Some populations may experience enhanced adverse air pollutant-associated health effects: children, the elderly, the obese, and those with underlying disease. In studies of subjects with a concurrent history of cardiovascular disease, air pollution exposure has been associated with greater inflammation, coagulation, and decreases in heart rate variability [16, 17]. In our previously conducted work in a cohort of coronary artery disease (CAD) patients, ambient ozone exposure was associated with changes in plasma metabolite levels [18]. This work suggests that metabolic processes may contribute to or mediate cardiovascular outcomes due to air pollutant exposure.

In order to expand upon this work we undertook a detailed panel study to assess whether there are alterations in several critical cardiovascular disease-associated biological pathways associated with acute ozone exposures. We recruited 13 volunteers with CAD to assess whether changes in endothelial function, fibrinolysis, inflammation, lipids, heart rate variability, and repolarization are observed following acute ozone exposure. In the interest of studying the effects of ozone in the context of particulate matter with a diameter of less than 2.5 μm (PM_2.5_) and to address potential confounding, we used a two-pollutant model to control for PM concentrations collected from central monitoring stations [19]. The results of this work may provide biological plausibility in support of the concept that ozone induces adverse cardiovascular effects in susceptible populations.

## Methods

### Study population and design

The source population for this study was the Duke University Medical Center CATHeterization GENetics (CATHGEN) cohort of nearly 10,000 individuals [20]. Duke University is located in central North Carolina in the city of Durham. To participate in CATHGEN, patients were between 40 and 75 years of age and had undergone a cardiac catheterization between 2001 and 2010 at a Duke University Hospital.

Starting with residents that resided within a reasonable commuting distance to the U.S. Environmental Protection Agency’s Human Studies Facility in Chapel Hill, NC, 448 letters were mailed out to participants enrolled in CATHGEN. The only exclusion criterion for not receiving a letter of invitation to participate was unstable angina and congestive heart failure. Fifty-four participants responded to the mailed letters. Of those, 15 participants meeting the severity criteria were enrolled for the current study. These volunteers were required to have a stable clinical status, documented coronary artery disease (> 75 occlusion in one major coronary vessel), a stable medication regimen over 3 months prior to enrollment, and an electrocardiogram demonstrating normal sinus rhythm. Exclusion criteria for the current study also included hematocrit < 34%, current smoking or smoking history within 1 year of study (defined as more than one pack of cigarettes in the past year), sustained cardiac arrhythmias, presence of a pacemaker or implanted cardioverter-defibrillator, systolic blood pressure (SBP) ≥ 150 mmHg or ≤ 90 mmHg or diastolic blood pressure (DBP) ≥ 100 mmHg, known vascular obstruction of the upper extremities, unstable angina, moderate to severe chronic pulmonary disease (as determined by spirometry demonstrating < 60% predicted value for forced vital capacity (FVC) or forced expiratory volume in 1s (FEV_1_) and including chronic obstructive pulmonary disease, pulmonary fibrosis, moderate to severe asthma, aortic stenosis), recent (past 6 months) myocardial infarction, cerebrovascular accident (i.e. stroke) or admission for heart failure, recent (past 6 months) vascular intervention/bypass surgery, or current pregnancy. Participants were also asked to refrain from vigorous exercise on study mornings and would be temporarily excluded from participation if they experienced a respiratory tract infection within the preceding 4 weeks or had a recent or recurring exposure to pollutants or irritants. All medications were evaluated by the study physician.

Each participant visited the U.S. Environmental Protection Agency’s Human Studies Facility for two consecutive days for up to 10 weeks, between May 2012 and April 2014. The Human Studies Facility is approximately 8 miles southwest of Duke University. Upon arrival on the first study day, the subject’s medical history was reviewed, vital signs were assessed, and the subject was outfitted with a Holter monitor which they would wear for the next 24 h. The following day, under fasting conditions, the subject was assessed for biomarkers present in blood, heart rate variability and repolarization measured by Holter monitoring, blood pressure, and endothelial function measured by brachial artery ultrasound and pulse wave analysis. To minimize day-of-week effects, each subject was always studied on the same 2 days of the week. Written informed consent was given by all participants prior to enrollment, and the study was approved by the Duke University Institutional Review Board, the University of North Carolina at Chapel Hill Institutional Review Board, and the U.S. Environmental Protection Agency (EPA) Human Protocols Office.

### Clinical measurements

#### Peripheral venous blood samples

Approximately 50 mL of venous blood was obtained from each subject. A portion of fresh blood was sent to a clinical laboratory (Lab Corp., Burlington, NC, USA) for analysis of differential blood cell counts, as well as quantification of blood lipids. The remaining blood samples were stored at −80 °C prior to analysis.

Commercially available multiplex kits (Meso Scale Diagnostics, Gaithersburg, MD) were used to quantify levels of C-reactive protein (CRP), serum amyloid A (SAA), soluble intercellular adhesion molecule (sICAM), soluble vascular adhesion molecule (sVCAM), interleukin 1-beta (IL-1β), interleukin-6 (IL-6), interleukin-8 (IL-8), and tumor necrosis factor-alpha (TNF-α) (SECTOR® Imager 2400, Meso Scale Diagnostics). All other assays (D-dimer, tissue plasminogen factor (tPA), von Willebrand factor (vWF), plasminogen activator inhibitor-1 (PAI-1), and plasminogen) were measured using MesoScale multi-array plates as per manufacturers’ instructions.

#### Brachial artery ultrasound

Based on the guidelines by Corretti et al. [21], brachial artery ultrasound (BAU) was measured to evaluate endothelial cell function using a 15 MHz transducer interfaced with an Acuson Sequoia 512 ultrasound machine (Siemens Healthcare, Malvern, PA, USA). As described earlier [22], resting blood pressure and the diameter of the brachial artery were measured at baseline, and the baseline diameter of the brachial artery (BAD) was also measured during reactive hyperemia for quantification of flow-mediated dilatation (FMD).

With the volunteer laying supine, a pneumatic tourniquet was placed around the right arm distal to the brachial artery. R-wave gated baseline images of the artery were acquired after 15 min. The cuff was then inflated to a pressure of 50 mmHg above the participant’s SBP for 5 min. The cuff was abruptly deflated to cause a hyperemia reaction. Images of the brachial artery were acquired for 90 s, stored in a digital format, and subsequently analyzed. Arterial diameter from the lumen-intimal interfaces of the proximal and distal walls was measured using customized software (Brachial Tools, Medical Imaging Applications, LLC, Coralville, IA, USA). Data from at least three consecutive end-diastolic frames were averaged for each baseline measurement and from at least three frames at maximal dilatation during reactive hyperemia. Changes in diameter caused by reactive hyperemia (endothelium-dependent vasodilatation) were expressed as a percent change in vessel diameter from their respective baselines.

#### Pulse wave analysis

Arterial elasticity was measured by the contour analysis of the arterial pressure waveform (pulse wave) using the HDI/PulseWave CR-2000 Research Cardiovascular Profiling system (Hypertension Diagnostics Inc., Eagan, MN, USA) as previously described [22]. Three assessments of arterial compliance were obtained and averaged. Measurements included the large-artery elasticity index (LAEI) and small-artery elasticity index (SAEI). The system gathered and analyzed a 30-s analog tracing of the radial artery waveforms digitized at 200 samples/s. A beat determination was made during the 30-s time period to determine systole, peak systole, onset of diastole, and end of diastole. Representative averaged waveforms of individual beats were analyzed using a parameter-estimating algorithm [23] to fit a multiplexed model [24]. Endothelial dysfunction is associated with decreasing elasticity indices.

#### Holter monitoring

Continuous ambulatory electrocardiograms (ECGs) were placed on each subject during the first day of each week’s two-day visit and were collected for 24 h periods using a Mortara H12+ 12-Lead ECG Recorder (Mortara Instrument Co., Milwaukee, WI) sampling at 180 Hz. During the second day, the subjects were asked to recline in a dark place, and data was collected for 30 min. A trained nurse manually inspected and edited the sequence of ECG complexes to ensure proper labeling. RR-intervals that were more or less than 20% of the previous RR-interval were defined as abnormally long or short intervals and were interpolated using Mortara algorithms. Subsequent heart rate variability (HRV) indices for both the time and frequency domains were calculated.

Time-domain measurements were calculated over the full 24 h span, while a 5 min segment during the end of the resting period was used for the calculation of the frequency-domain and repolarization indices. Time-domain measurements included standard deviation of the normal-to-normal (SDNN) and root-mean squared of successive differences (rMSSD). Frequency-domain measurements included low frequency (LF, 0.04–0.15 Hz), high frequency (HF, 0.15–0.40 Hz), low-to-high frequency power (LF:HF), and the sum of the power spectrum density (PSD).

Markers of cardiac repolarization were assessed by measuring the QT interval, which is it the measurement from the beginning of the QRS complex to the end of the T wave; we corrected the QT interval for heart rate (QTc). T wave complexity was measured in each beat by principal component analysis based on all 12 leads and averaged. QRS complexity and P wave complexity were calculated with Mortara software.

### Air pollution and meteorological measurements

Daily 24 h measurements of ozone and PM_2.5_ were calculated from hourly pollutant data averaged between 9 AM to 8 AM; this data was obtained from a central air monitoring station (Millbrook) located approximately 44 km (27 miles) from the EPA Human Studies Facility and operated by the State of North Carolina. Two visits used pollutant data from a different central monitor (Durham Armory) due to missing values; this location was approximately 18 km (11 mi) from the EPA Human Studies Facility. Concentrations were obtained for each clinic day, as well as for 4 days prior. Daily temperature, relative humidity, and pressure were also obtained from the Millbrook central monitoring station.

### Statistical analysis

The study was conducted as a panel study with four to ten repeated measurements per participant. Thus, every subject acted as his/her own control, limiting the need for an adjustment for subject characteristics. Data were analyzed using the R statistical package (Version 3.3.0) using both a single (ozone) and two-pollutant (ozone and PM_2.5_) model. For the analyses, we used additive mixed models with a random subject effect that diminished the need for an adjustment for subject characteristics. Daily temperature and relative humidity corresponding to the lag of the pollutant were selected as covariates a priori, and we adjusted for seasonal trends using a natural spline. To determine the additional benefit of including a five-day average of barometric pressure as a covariate, models including and excluding the pressure term were compared on the basis of Akaike Information Criterion (AIC) and changes to the ozone coefficient. For this work, the ozone exposure was considered either as an immediate (lag 0), delayed (lag 1 to 4), or cumulative (5 day moving average, 5dMA) linear effect, similar to our previously published work [25]. All outcomes were log transformed prior to analysis, are reported as percent change from the mean of the measured outcome per unit interquartile range (IQR) of exposure, and statistical significance was set at *p* < 0.05 for the percent change from the mean of the measured outcome per unit IQR of exposure.

## Results

**Fig. 1:**
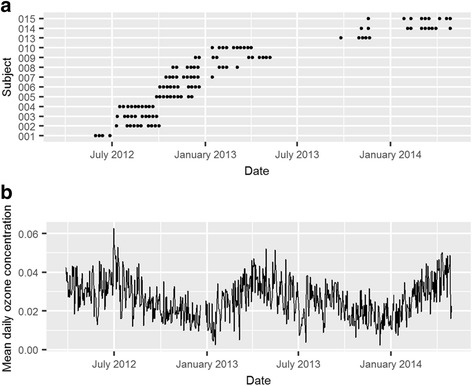
Patient visits and mean daily ozone concentrations (ppm) between May 30, 2012-April 29, 2014. **a** Data points represent the days the patients visited the Human Studies Facility. **b** Daily 24-h ozone (ppm) concentrations (9 AM to 8 AM) calculated from the Millbrook central monitor

Of the 15 participants that were recruited for this study, two completed less than three study sessions and were not included in the final analysis (Table 1). The subjects’ ages ranged from 53 to 68 years. Although inclusion in the study was open to both men and women, only men responded to advertisements and successfully passed the screening criteria. Most of the participants were taking medication: six subjects reported taking medication for diabetes, nine subjects were taking beta blockers, and twelve were taking HMG CoA reductase inhibitors (statins). Of the 13 subjects, five had experienced a previous myocardial infarction and nine had hypertension. Although subjects were excluded for being current smokers or smoking more than one pack of cigarettes 1 year before enrolling in this study, seven participants had a past history of smoking. Nine participants completed ten visits, two participants completed nine visits, one participant completed five visits, and one participant completed four visits. Altogether there were 117 exposure days analyzed (Fig. 1a).

**Table 1:** Participant characteristics (*n* = 13)

Characteristics (n = 13)	Value
Age (years)	63 (53–68)
Males	13 (100%)
Race	
Caucasian	10 (77%)
Black	3 (23%)
Current health status	
BMI (kg/m^2^)	31 (26–38)
Systolic blood pressure	130 (102–141)
Diastolic blood pressure	77 (65–90)
Asthma	1 (8%)
Current medication use	
Diabetes medication	6 (46%)
Beta blockers	9 (69%)
Statins	12 (92%)
Past health status	
Previous MI	5 (38%)
Previous hypertension	9 (69%)
Past smokers	7 (54%)

Values expressed as either mean (range) or number (%). *BMI* body mass index, *MI* myocardial infarction

Air pollution and meteorological measurements are shown in Table 2. Ozone concentrations during the entire study period (May 30, 2012-April 29, 2014) ranged from 0.002 to 0.063 ppm, with an interquartile range of 0.014 ppm and a mean value of 0.026 ± 0.010 ppm. Ozone concentrations at the Millbrook and Armory central monitoring stations were highly correlated (Spearman correlation coefficient = 0.92; *p* < 0.0001). PM_2.5_ mass concentrations during the study period ranged from 1.0 to 28.2 μg/m^3^, with an interquartile range of 5.4 μg/m^3^ and a mean value of 10.9 ± 4.5 μg/m^3^. Ozone and PM_2.5_ concentrations were not significantly correlated (Spearman correlation coefficient = 0.05; *p* = 0.19). Temperature and humidity fluctuations during the study period ranged from 18.4 to 91.1 °F and 26.5 to 96.3%, respectively. In this paper, we describe the association of ozone with various cardiovascular outcomes, using both a one- (ozone) and two-pollutant (ozone and PM_2.5_) model. The results for all endpoints are shown in Additional file 1: (Table S1). Associations between PM_2.5_ and cardiovascular outcomes will be described elsewhere. Figure 1 shows the daily ozone concentrations during the nearly two-year duration of the study and at the date of each subject’s clinical visits, which were spread out across various seasons to get a range of ozone concentrations for this work. The maximum observed ozone and PM_2.5_ concentrations during the study duration were below the EPA National Ambient Air Quality Standards, which are 0.070 ppm for ozone (8 h) and 35 μg/m^3^ for PM_2.5_ (24 h).

**Table 2:** Average daily ozone concentrations during study period (May 30, 2012-April 29, 2014)

	Mean ± SD	Minimum	25th percentile	Median	75th percentile	Maximum	IQR
Ozone (ppm)	0.026 ± 0.010	0.002	0.019	0.025	0.033	0.063	0.014
PM_2.5_ (μg/m^3^)	10.9 ± 4.5	1.0	7.8	9.9	13.2	28.2	5.4
Temperature (°F)	59.1 ± 16.0	18.4	45.2	60.1	74.0	91.1	28.8
Relative humidity (%)	67.9 ± 14.2	26.5	57.9	70.0	78.4	96.3	20.5
Barometric pressure (hPa)	1018.2 ± 6.0	1001.4	1014.0	1018.0	1021.9	1036.4	7.9

*IQR* interquartile range, *PM* particulate matter, *SD* standard deviation

**Fig. 2:**
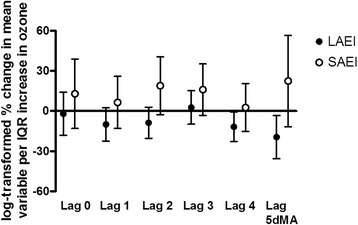
Percent changes of elasticity indices with ambient ozone concentrations. Effect estimates (95% CI) were log-transformed, correspond to changes per IQR of ozone, and were adjusted for season, temperature, and humidity. IQR = interquartile range; LAIE = large artery elasticity index; SAEI = small artery elasticity index; 5dMA = 5 day moving average

**Fig. 3:**
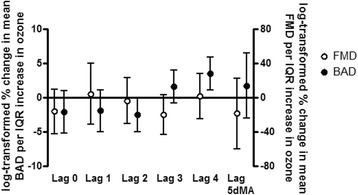
Percent changes of endothelial function with ambient ozone concentrations. Effect estimates (95% CI) were log-transformed, correspond to changes per IQR of ozone, and were adjusted for season, temperature, and humidity. Effect estimates for FMD were also adjusted for the 5dMA barometric pressure. IQR = interquartile range; FMD = flow-mediated dilatation; BAD = baseline artery diameter; 5dMA = 5 day moving average

Large artery elasticity index (LAEI) decreased with increasing ozone concentrations (Fig. 2). There was a significant 19.5% decrease for the 5 day moving average (95% confidence intervals (CI) = −34.0, −1.7; *p* = 0.03), and a borderline significant 11.7% decrease with a lag of 4 days (95% CI = −22.1, 0.0; *p* = 0.05). LAEI is a measure of arterial compliance, defined as the ability of an artery to expand and recoil with cardiac pulsation and relaxation. A decrease in compliance results in a stiffening of the artery and is a risk factor for atherosclerosis. For SAEI, there was a trend for an increase with lags of 2 and 3 days. There was a 2.5% decrease in the baseline diameter of the brachial artery (BAD) associated with ozone with a 2 day lag (95% CI = −5.0, 0.1; *p* = 0.06). This was counterbalanced with a significant 3.5% increase with a 4 day lag (95% CI = 1.2, 5.9; *p* < 0.01; Fig. 3). No significant changes were observed for FMD (Additional file 1: Table S1). There was also a 3.3% marginally significant decrease in diastolic blood pressure with a 2 day lag (95% CI = −6.6, 0.2; *p* = 0.07). Similar decreases in blood pressure associated with ozone exposures have been reported by others [26].

**Fig. 4:**
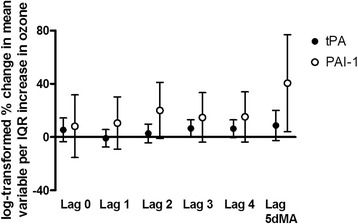
Percent changes of clotting and fibrinolysis factors with ambient ozone concentrations. Effect estimates (95% CI) were log-transformed, correspond to changes per IQR of ozone, and were adjusted for season, temperature, and humidity. IQR = interquartile range; tPA = tissue plasminogen factor; PAI-1 = plasminogen activator inhibitor-1; 5dMA = 5 day moving average

Factors attributed to clotting and fibrinolysis included tissue plasminogen factor (tPA), plasminogen activator inhibitor-1 (PAI-1), von Willebrand factor (vWF), plasminogen, and D-dimer. Ozone was associated with a 6.6% increase in tPA with a 3 day lag (95% CI = 0.4, 13.2; *p* = 0.04), and a near-significant 6.3% increase with a 4 day lag (95% CI = −0.1, 13.1; *p* = 0.05; Fig. 4), per IQR of ozone. A 20.0% increase in PAI-1 with a 2 day lag (95% CI = 0.8, 42.8; *p* = 0.04), and a 40.5% increase with a 5 day moving average were also observed (95% CI = 8.7, 81.6; *p* = 0.01); there were near-significant increases with 3 (95% CI = −2.3, 35.0; *p* = 0.09) and 4 (95% CI = −2.1, 35.6; *p* = 0.09) day lags. PAI-1 and tPA are associated the fibrinolysis pathway. No significant changes were observed for von Willebrand factor, plasminogen, and D-dimer (Additional file 1: Table S1).

**Fig. 5:**
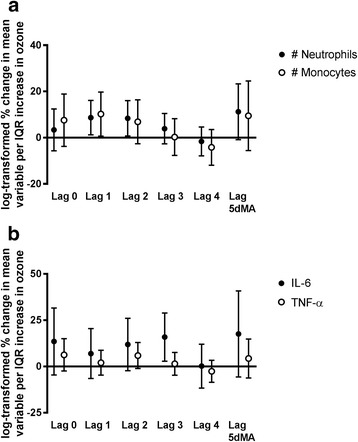
Percent changes of inflammatory factors with ambient ozone concentrations. Effect estimates (95% CI) were log-transformed, correspond to changes per IQR of ozone, and were adjusted for season, temperature, and humidity. **a** Percent changes in the number of neutrophils and monocytes; **b** Percent changes in IL-6 and TNF-α. IQR = interquartile range; IL = interleukin; TNF = tumor necrosis factor; 5dMA = 5 day moving average

The number of neutrophils and monocytes, as well as the concentrations of IL-6, IL-8, TNF-α, CRP, SAA, sICAM, and sVCAM, were measured. Per IQR of ozone, an 8.7% increase in the number of neutrophils was observed with a 1 day lag (95% CI = 1.5, 16.4; *p* = 0.02), an 8.4% increase was observed with a 2 day lag (95% CI = 1.0, 16.3; *p* = 0.03), and a near-significant 11.2% increase was observed with the 5 day moving average (95% CI = −0.2, 23.9; *p* = 0.05; Fig. 5). Ozone was also associated with a 10.2% increase in the number of monocytes following 1 day lag (95% CI = 1.0, 20.1; *p* = 0.03). For IL-6, increases in 11.9% (95% CI = −1.3, 27.0; *p* = 0.08) and 15.9% (95% CI = 3.6, 29.6; *p* = 0.01) per IQR of ozone were observed at 2 and 3 day lags, respectively; and a 5.9% increase in TNF-α was found following a 2 day lag (95% CI = −0.9, 13.2; *p* = 0.09; Fig. 5). Finally, a 9.6% decrease in sVCAM was associated with increases 2 days prior to the collection of biological specimens (95% CI = −17.2, −1.2; *p* = 0.03). No significant associations with ozone were observed for IL-8, CRP, SAA, or sICAM (Additional file 1: Table S1).

As shown in Additional file 1: (Table S1), ozone was not significantly associated with changes in heart rate variability as measured by the standard deviation of the beat-to-beat interval (SDNN), root-mean squared of successive differences between adjacent NN intervals (rMSSD), low frequency domain (LF), high frequency domain (HF), and sum of the power spectrum density (PSD). Ozone was also not associated with measurements of repolarization including the duration of the QT interval (QTc), or complexity of the P-wave, T-wave, or QRS. We also found no associations between ozone and changes in lipids, including cholesterol, triglycerides, high density lipoprotein (HDL), or low density lipoprotein (LDL).

To determine if the associations observed with ozone were confounded by PM_2.5_, a two-pollutant model was used (Additional file 2: Table S2). For those outcomes that were associated with ozone in the single pollutant model, minimal (less than 10%) changes were observed in the effect estimates when PM_2.5_ was included in the two-pollutant model, suggesting that the effects observed for ozone were specific to ozone and independent of PM_2.5_. In some cases, confidence intervals were slightly widened in the two-pollutant model, while in others they were slightly narrowed. Because of the limited sample size in this study, effect modification by age, gender, medication, and others could not be determined.

## Discussion

In this panel study of a population with stable CAD, we observed associations between ambient ozone concentrations and changes in biological pathways involved in inflammation, fibrinolysis, and endothelial cell function. These changes were still evident when controlling for PM_2.5_ concentrations, suggesting that these results were independent of PM_2.5_ exposures.

Endothelium-derived mediators as well as vascular smooth muscle function regulate vascular tone structure, which can influence arterial stiffness and diameter. Arterial stiffness is the reduced ability of an artery to contract and expand during pressure changes [27]. One of the parameters used to describe arterial stiffness is arterial compliance, which is the measure of volume changes in a vessel in response to changes in arterial pressure [27]. Studies have found inverse associations between arterial compliance and age, glucose levels, smoking, hypertension, and metabolic syndrome [28, 29]. In the current study, we observed associations between ozone and a decrease in LAEI, indicating reduced arterial compliance. Past studies looking at other measures of arterial compliance have also reported negative associations with ozone exposure in elderly males as part of the VA Normative Aging Study Cohort [30] and in healthy adults [31]. In addition to LAEI, we observed an association between ozone and a decreased diameter of the brachial artery. Vasoconstriction caused by arterial narrowing can increase blood pressure, which could result in adverse outcomes in susceptible populations such as those with hypertension.

In this current work, we also found associations between ozone and increased levels of two critical components of the fibrinolysis pathway: tPA and PAI-1. The fibrinolytic pathway degrades blood clots that are formed during the normal course of living [32], and any process that inhibits this pathway increases the risk for adverse events associated with coagulation. When bound with tPA, plasminogen is converted to plasmin, which then degrades fibrin and dissolves clots. PAI-1 inhibits tPA, and increasing concentrations of PAI-1 are associated with decreases in fibrinolysis. Concentrations of PAI-1 in plasma are much higher than tPA, and assays quantifying tPA levels generally reflect the concentrations of tPA complexed with PAI-1 [33]. For this reason, increasing concentrations of tPA also can indicate reduced fibrinolysis [33]. Similar increases in tPA were observed in a previously conducted controlled human exposure study of ozone and clean air in healthy, young participants [11]. In a panel study of 76 young, healthy students in Taipei, increases in PAI-1 and tPA were found with 1- to 3- day averages of ambient ozone concentrations in both a single- and multi-pollutant model [34]. Taken as a whole, our data suggest that ozone can negatively impact the fibrinolysis pathway.

A large body of research has shown associations between ozone and cellular and soluble makers of pulmonary inflammation [35, 36]. In a previously conducted controlled ozone study, increases in the systemic inflammatory markers IL-1β, IL-6, and TNF-α were observed 24 h following ozone exposures compared to baseline values [12]. In a separate controlled study, plasma levels of IL-6 were elevated in both obese and non-obese non-smoking women after acute ozone exposures [37]. When 45 non-smoking adults enrolled in a panel study to assess the association of ambient air pollution and systemic inflammation, positive associations between IL-6 and ozone were observed [38]. In this current work, we report an association between ozone and increased levels of cellular and soluble markers of vascular inflammation, as well as associations between ozone and increased numbers of monocytes, neutrophils, and two markers of inflammation: IL-6 and TNF-α. Clinically, increases in plasma IL-6 and TNF-α have been associated with cardiovascular disease and disease outcomes [39].

Our findings in humans with CAD corroborate some experimental data from animals exposed to air pollution. When rats were exposed to 0.50 ppm of ozone for 5 h/day for 2 days, increases in PAI-1 were observed [40]. However, in contrast, Farraj et al. [41] observed significant changes in heart rate variability parameters and blood lipids in spontaneously hypertensive rats exposed to various ozone concentrations; these changes were only observed after high ozone exposures (0.8 ppm), and no changes in heart rate variability were found following lower exposures (0.2 ppm). Therefore, it is possible that we did not observe alterations in heart rate variability due to the relatively low ozone concentration observed in the ambient environment compared with those used in laboratory studies.

We observed that several of the biological pathways disrupted by PM_2.5_ exposures may also be altered by ozone exposures. For example, we previously reported decreased levels of LAEI to be associated with PM_2.5_ in type 2 diabetics [22]. Additionally, increases in tPA [42] and systemic pro-inflammatory cytokines [43] are associated with particle exposures.

In contrast with studies looking at cardiovascular changes or blood lipids associated with PM_2.5_ exposures [25, 44], we did not observe associations between ozone and any marker of heart rate variability or repolarization, or in blood lipids. It is possible that medication use prevented us from seeing these effects. For example, 70% of the participants were taking beta blocker medication which is known to interfere with HRV analysis [45], and a similar proportion were taking statin medications, which may impact air pollution-induced increases in blood lipids.

Lag effects may differ between ozone and PM_2.5_ as well. In a previous panel study of diabetic patients, we observed an association between PM_2.5_ and inflammatory changes at lags 2–3, but endothelial function changes occurred with a 0 day lag [22, 25]. In the current study, we did not observe rapid changes (i.e. those occurring with a 0 or 1 day lag) associated with ozone, with the exception being systemic inflammatory mediators. This is consistent with observations of rapid responses in human systemic inflammatory markers in laboratory-controlled conditions [11]. Similarly, Bind et al. [46] observed ozone-induced responses in pro-inflammatory mediators as early as 4 and 24 h, which then decreased in intensity at later time points.

There are several strengths in this study. Unlike prior studies, we examined a significantly at-risk population – those with active CAD. Further, given the recent interest in examining the effects of multiple pollutants simultaneously, we used a two-pollutant model to adjust for PM_2.5_ concentrations, observing ozone effects independent of PM_2.5_. We also studied the time-course of effects at several temporal lags relative to exposure. Last, via the repeated measures study design, we accounted for both between- and within-subject variability.

There were also several limitations. It is possible that our study may suffer from selection bias; inclusion in the CATHGEN cohort could be as a result of referral bias, enrollment biases, and those related to socioeconomic status and other unmeasured confounders. We also used central monitoring stations for our exposures, which may result in exposure misclassification. All of the subjects were taking at least one cardiovascular disease medication, which could have affected our ability to detect ozone-associated differences. Further, given the relatively small sample size, we were not able to assess for effect modification such as genotype, body mass index, and medication [22, 25].

## Conclusions

In this panel study of ambient air pollution exposure in a population with extant coronary heart disease, we observed ozone-associated alterations in several pathways associated with cardiovascular morbidity and mortality: fibrinolysis, systemic inflammation, and vascular reactivity. The effects remained strong after controlling for PM_2.5_ concentrations. Future work will compare these effects with effects associated with PM_2.5_ in the same cohort. This study contributes new information regarding the mechanisms underlying the effects of ozone on cardiovascular risk. The observed effects were found at ambient concentrations below the EPA National Ambient Air Quality Standards for both ozone and PM_2.5_.

## Additional files

Additional file 1: Table S1. Percent changes of measured factors with ambient ozone concentrations. Effect estimates (95% CI) were log-transformed, correspond to changes per IQR of ozone, and were adjusted for season, temperature, and humidity. Effect estimates for SumPSD, LF:HF, LF, HF, FMD, and CRP were also adjusted for the 5dMA barometric pressure. LAIE = large artery elasticity index; SAEI = small artery elasticity index; FMD = flow-mediated dilatation; BAD = baseline artery diameter; SBP = systolic blood pressure; DBP = diastolic blood pressure; tPA = tissue plasminogen factor; PAI-1 = plasminogen activator inhibitor-1; vWF = von Willebrand factor; IL = interleukin; TNF = tumor necrosis factor; CRP = C-reactive protein; SAA = serum amyloid A; sICAM = soluble intercellular adhesion molecule; sVCAM = soluble vascular adhesion molecule; HDL = high density lipoprotein; LDL = low density lipoprotein; LF = low frequency; HF = high frequency; PSD = power spectrum density; SDNN = standard deviation of the normal-to-normal; rMSSD = root-mean squared of successive differences. *p value < 0.10 for the percent change from the mean of the measured outcome per unit IQR of exposure, **p value < 0.05 for the percent change from the mean of the measured outcome per unit IQR of exposure. (DOCX 21 kb)

Additional file 2: Table S2. Percent changes of measured factors with ambient ozone and PM_2.5_ concentrations using a two-pollutant model. Effect estimates (95% CI) were log-transformed, correspond to changes per IQR of the corresponding pollutants, and were adjusted for season, temperature, and humidity. Effect estimates for FMD were also adjusted for the 5dMA barometric pressure. LAIE = large artery elasticity index; SAEI = small artery elasticity index; FMD = flow-mediated dilatation; BAD = baseline artery diameter; IL = interleukin; TNF = tumor necrosis factor; tPA = tissue plasminogen factor; PAI-1 = plasminogen activator inhibitor-1. *p value < 0.10 for the percent change from the mean of the measured outcome per unit IQR of exposure, **p value < 0.05 for the percent change from the mean of the measured outcome per unit IQR of exposure. (DOCX 21 kb)

## Abbreviations

5dMA: 5 day moving average
AIC: Akaike Information Criterion
BAD: Diameter of the brachial artery
BAU: Brachial artery ultrasound
CAD: Coronary artery disease
CATHGEN: CATHeterization GENetics
CI: Confidence intervals
CRP: C-reactive protein
DBP: Diastolic blood pressure
ECG: Electrocardiograms
EPA: Environmental Protection Agency
FEV_1_: Forced expiratory volume in 1s
FMD: Flow-mediated dilatation
FVC: Forced vital capacity
HDL: High density lipoprotein
HF: High frequency
HRV: Heart rate variability
IL-1β: Interleukin 1-beta
IL-6: Interleukin-6
IL-8: Interleukin-8
IQR: Interquartile range
LAEI: Large-artery elasticity index
LDL: Low density lipoprotein
LF: Low frequency
PAI-1: Plasminogen activator inhibitor-1
PM: Particulate matter
PSD: Power spectrum density
QTc: QT interval corrected
rMSSD: Root-mean squared of successive differences
SAA: Serum amyloid A
SAEI: Small-artery elasticity index
SBP: Systolic blood pressure
SDNN: Standard deviation of the normal-to-normal
sICAM: Soluble intercellular adhesion molecule
sVCAM: Soluble vascular adhesion molecule
TNF-α: Tumor necrosis factor-alpha
tPA: Tissue plasminogen factor
vWF: Von Willebrand factor
