# Supplementary material for: Ozone exposure is associated with acute changes in inflammation, fibrinolysis, and endothelial cell function in coronary artery disease patients
Source: Environ Health. 2017 Nov 21;16:126. doi: 10.1186/s12940-017-0335-0 (PMC5697214; doi:10.1186/s12940-017-0335-0)
Supplement: Supplementary file 1 — Percent changes of measured factors with ambient ozone concentrations. Effect estimates (95% CI) were log-transformed, correspond to changes per IQR of ozone, and were adjusted for season, temperature, and humidity. Effect estimates for SumPSD, LF:HF, LF, HF, FMD, and CRP were also adjusted for the 5dMA barometric pressure. LAIE = large artery elasticity index; SAEI = small artery elasticity index; FMD = flow-mediated dilatation; BAD = baseline artery diameter; SBP = systolic blood pressure; DBP = diastolic blood pressure; tPA = tissue plasminogen factor; PAI-1 = plasminogen activator inhibitor-1; vWF = von Willebrand factor; IL = interleukin; TNF = tumor necrosis factor; CRP = C-reactive protein; SAA = serum amyloid A; sICAM = soluble intercellular adhesion molecule; sVCAM = soluble vascular adhesion molecule; HDL = high density lipoprotein; LDL = low density lipoprotein; LF = low frequency; HF = high frequency; PSD = power spectrum density; SDNN = standard deviation of the normal-to-normal; rMSSD = root-mean squared of successive differences. *p value < 0.10 for the percent change from the mean of the measured outcome per unit IQR of exposure, **p value < 0.05 for the percent change from the mean of the measured outcome per unit IQR of exposure. (DOCX 21 kb) [file 12940_2017_335_MOESM1_ESM.docx]

|  | **Lag 0** | **Lag 1** | **Lag 2** | **Lag 3** | **Lag 4** | **5 day average** |
| --- | --- | --- | --- | --- | --- | --- |
| **ENDOTHELIAL** **FUNCTION MEASUREMENTS** | | | | | | |
| LAEI | -2.2 (-17.0, 15.2) | -10.0 (-21.6, 3.2) | -8.8 (-19.6, 3.6) | 2.6 (-9.2, 15.9) | -11.7 (-22.1, 0.0)* | -19.5 (-34.0, -1.7)** |
| SAEI | 12.9 (-10.2, 41.8) | 6.4 (-11.3, 27.7) | 18.9 (-0.7, 42.4)* | 16.0 (-1.7, 36.8)* | 2.6 (-13.7, 22.1) | 22.4 (-7.0, 61.1) |
| FMD | -16.0 (-38.1, 14.1) | 4.5 (-25.4, 46.5) | -3.7 (-26.9, 27.0) | -19.8 (-39.6, 6.3) | 1.7 (-21.1, 31.0) | -18.6 (-49.9, 32.1) |
| BAD | -2.1 (-5.1, 1.0) | -1.9 (-4.9, 1.2) | -2.5 (-5.0, 0.1)* | 1.6 (-0.7, 4.0) | 3.5 (1.2, 5.9)** | 1.7 (-2.9, 6.6) |
| SBP | 2.3 (-2.0, 6.7) | -0.1 (-3.3, 3.4) | 0.0 (-3.3, 3.4) | -1.7 (-4.6, 1.3) | 1.9 (-1.3, 5.1) | 1.4 (-3.5, 6.5) |
| DBP | 2.3 (-2.1, 6.9) | -1.8 (-5.2, 1.7) | -3.3 (-6.6, 0.2)* | -0.5 (-3.6, 2.6) | 2.4 (-1.0, 5.8) | -0.4 (-5.5, 5.0) |
| **CLOTTING/FIBRINOLYSIS FACTORS** | | | | | | |
| tPA | 5.4 (-3.1, 14.7) | -0.9 (-7.2, 5.9) | 2.7 (-4.0, 10.0) | 6.6 (0.4, 13.2)** | 6.3 (-0.1, 13.1)* | 8.8 (-2.0, 20.7) |
| PAI-1 | 8.2 (-12.8, 34.3) | 10.6 (-7.3, 32.0) | 20.0 (0.8, 42.8)** | 14.8 (-2.3, 35.0)* | 15.2 (-2.1, 35.6)* | 40.5 (8.7, 81.6)** |
| Plasminogen | 0.7 (-8.2, 10.6) | -2.4 (-9.3, 5.0) | -0.66 (-7.8, 7.0) | 2.8 (-4.0, 10.0) | 2.3 (-4.6, 9.6) | 2.3 (-8.8, 14.6) |
| vWF | -3.0 (-13.5, 8.7) | 5.4 (-3.16, 14.6) | 3.0 (-5.6, 12.4) | 4.6 (-3.3, 13.1) | 1.3 (-6.5, 9.8) | 10.0 (-4.1, 26.1) |
| D dimer | 1.1 (-18.5, 25.3) | 0.5 (-15.6, 19.8) | -8.7 (-23.6, 9.1) | 2.0 (-13.0, 19.7) | -9.4 (-23.0, 6.5) | -5.9 (-28.6, 24.0) |
| **INFLAMMATION MARKERS** | | | | | | |
| # Neutrophils | 3.4 (-5.3, 12.8) | 8.7 (1.5, 16.4)** | 8.4 (1.0, 16.3)** | 3.9 (-2.5, 10.7) | -1.6 (-7.7, 4.8) | 11.2 (-0.2, 23.9)* |
| # Monocytes | 7.6 (-3.2, 19.5) | 10.2 (1.0, 20.1)** | 6.9 (-2.2, 16.8) | 0.3 (-7.4, 8.5) | -4.2 (-11.6, 3.9) | 9.5 (-4.5, 25.6) |
| IL-6 | 13.5 (-3.14 33.1) | 7.0 (-5.6, 21.3) | 11.9 (-1.3, 27.0)* | 15.9 (3.6, 29.6)** | 0.2 (-10.9, 12.8) | 17.6 (-3.4, 43.1) |
| IL-8 | -6.5 (-36.1, 36.9) | 13.2 (-16.5, 53.4) | -9.2 (-32.50 22.1) | 19.7 (-8.7, 57.0) | -10.4 (-32.3, 18.7) | -1.3 (-38.7, 58.9) |
| TNF-α | 6.3 (-2.1, 15.4) | 2.1 (-4.5, 9.0) | 5.9 (-0.9, 13.2)* | 1.4 (-4.5, 7.8) | -2.6 (-8.4, 3.5) | 4.3 (-5.7, 15.4) |
| CRP | -1.5 (-42.3, 68.2) | 3.2 (-32.6, 58.2) | -6.4 (-38.9, 43.4) | 6.8 (-28.7, 59.9) | 2.3 (-30.5, 50.4) | 2.5 (-49.0, 106.0) |
| SAA | -3.5 (-30.1, 33.2) | 11.0 (-13.9, 43.1) | 18.3 (-8.8, 53.3) | 1.8 (-19.6, 28.9) | 3.7 (-18.4, 31.7) | 27.8 (-14.1, 90.2) |
| sICAM | 4.1 (-5.8, 14.9) | 0.3 (-7.2, 8.3) | -5.7 (-12.9, 2.0) | 0.5 (-6.4, 8.0) | 3.0 (-4.3, 10.8) | 4.5 (-7.5, 18.1) |
| sVCAM | 1.0 (-9.8, 13.0) | -1.4 (-9.6, 7.6) | -9.6 (-17.2, -1.2)** | -1.4 (-9.1, 6.9) | 4.5 (-3.8, 13.4) | -0.9 (-13.7, 13.9) |
| **BLOOD LIPIDS** | | | | | | |
| Cholesterol | -0.4 (-5.0, 4.3) | -2.0 (-5.7, 1.8) | 0.9 (-3.1, 5.0) | -0.7 (-4.0, 2.7) | 0.2 (-3.2, 3.8) | -0.3 (-6.4, 6.2) |
| HDL | -3.1 (-6.9, 0.9) | -2.6 (-5.8, 0.6) | 2.2 (-1.3, 5.8) | -2.3 (-5.2, 0.6) | -0.6 (-3.6, 2.5) | -3.5 (-8.7, 1.9) |
| LDL | 1.6 (-6.5, 10.5) | -1.5 (-7.9, 6.1) | 1.1 (-5.6, 8.4) | -0.4 (-6.2, 5.7) | -0.5 (-5.5, 6.8) | 1.1 (-9.3, 12.8) |
| Triglycerides | 0.53 (10.8, 13.3) | 1.1 (-8.3, 11.4) | -1.6 (-11.3, 9.2) | -0.1 (-8.7, 9.3) | -4.1 (-12.6, 5.4) | 3.2 (-11.8, 20.9) |
| **HEART RATE VARIABILITY MEASUREMENTS** | | | | | | |
| LF:HF | -7.3 (-42.7, 49.9) | 18.0 (-22.0, 79.1) | -15.2 (-42.4, 25.0) | -9.8 (-38.4, 32.1) | 1.6 (-30.1, 47.6) | -25.0 (-59.6, 39.1) |
| Sum PSD | 13.5 (-30.4, 85.1) | 0.8 (-34.0, 54.0) | -8.9 (-39.2, 36.6) | 17.4 (-20.6, 73.5) | 5.8 (-27.8, 55.1) | -11.3 (-53.0, 67.6) |
| HF | 2.0 (-38.4, 68.8) | 11.3 (-27.7, 71.5) | -17.1 (-45.0, 24.8) | 1.2 (-31.7, 49.8) | 9.8 (-24.9, 60.6) | -21.9 (-58.2, 46.0) |
| LF | 8.4 (-32.6, 74.2) | -7.7 (-37.8, 36.9) | 2.5 (-30.9, 52.0) | 16.9 (-19.8, 70.6) | 3.8 (-27.6, 48.8) | 5.1 (-44.7, 99.8) |
| SDNN | 0.2 (-11.0, 12.8) | -2.7 (-12.1, 7.7) | 1.0 (-8.6, 11.5) | 0.0 (-9.1, 10.0) | 4.9 (-4.7, 15.5) | -6.2 (-18.9, 8.5) |
| rMSSD | 5.7 (-12.3, 27.3) | 2.0 (-12.7, 19.1) | 4.0 (-10.4, 20.8) | 1.3 (-12.4, 17.1) | 5.0 (-9.3, 21.6) | -4.9 (-23.6, 18.4) |
| **REPOLARIZATION MEASUREMENTS** | | | | | | |
| P complexity | -0.2 (-9.0, 9.5) | -0.9 (-8.2, 7.1) | 1.2 (-6.4, 9.5) | 1.5 (-5.4, 8.9) | -3.8 (-10.4, 3.3) | -2.9 (-13.6, 19.) |
| QRS complexity | 2.9 (-5.6, 12.2) | -1.1 (-7.7, 6.0) | -2.4 (-9.0, 4.7) | 0.3 (-5.9, 6.9) | 3.2 (-3.4, 10.2) | -0.2 (-10.3, 11.2) |
| T complexity | -6.3 (-15.8, 4.4) | 2.7 (-6.0, 12.4) | -4.7 (-13.0, 4.4) | 0.6 (-7.6, 9.6) | -1.9 (-10.0, 6.9) | 2.6 (-10.3, 17.3) |
| QT | -0.8 (-2.0, 0.4) | 0.5 (-0.6, 1.5) | 0.4 (-0.6, 1.4) | 0.1 (-0.8, 1.0) | -0.5 (-1.4, 0.5) | -0.6 (-2.1, 0.8) |
| QTCdyn | 0.2 (-0.7, 1.1) | 0.6 (-0.1, 1.4) | 0.2 (-0.6, 0.9) | -0.2 (-0.9, 0.4) | -0.9 (-1.6, -0.3)** | -0.3 (-1.3, 0.8) |

**Additional Table 1. Percent changes of measured factors with ambient ozone concentrations.** Effect estimates (95% CI) were log-transformed, correspond to changes per IQR of ozone, and were adjusted for season, temperature, and humidity. Effect estimates for SumPSD, LF:HF, LF, HF, FMD, and CRP were also adjusted for the 5dMA barometric pressure. LAIE = large artery elasticity index; SAEI = small artery elasticity index; FMD = flow-mediated dilatation; BAD = baseline artery diameter; SBP = systolic blood pressure; DBP = diastolic blood pressure; tPA = tissue plasminogen factor; PAI-1 = plasminogen activator inhibitor-1; vWF = von Willebrand factor; IL = interleukin; TNF = tumor necrosis factor; CRP = C-reactive protein; SAA = serum amyloid A; sICAM = soluble intercellular adhesion molecule; sVCAM = soluble vascular adhesion molecule; HDL = high density lipoprotein; LDL = low density lipoprotein; LF = low frequency; HF = high frequency; PSD = power spectrum density; SDNN = standard deviation of the normal-to-normal; rMSSD = root-mean squared of successive differences. *p value < 0.10 for the percent change from the mean of the measured outcome per unit IQR of exposure, **p value < 0.05 for the percent change from the mean of the measured outcome per unit IQR of exposure.
