# Supplementary material for: Ozone exposure is associated with acute changes in inflammation, fibrinolysis, and endothelial cell function in coronary artery disease patients
Source: Environ Health. 2017 Nov 21;16:126. doi: 10.1186/s12940-017-0335-0 (PMC5697214; doi:10.1186/s12940-017-0335-0)
Supplement: Supplementary file 2 — Percent changes of measured factors with ambient ozone and PM2.5 concentrations using a two-pollutant model. Effect estimates (95% CI) were log-transformed, correspond to changes per IQR of the corresponding pollutants, and were adjusted for season, temperature, and humidity. Effect estimates for FMD were also adjusted for the 5dMA barometric pressure. LAIE = large artery elasticity index; SAEI = small artery elasticity index; FMD = flow-mediated dilatation; BAD = baseline artery diameter; IL = interleukin; TNF = tumor necrosis factor; tPA = tissue plasminogen factor; PAI-1 = plasminogen activator inhibitor-1. *p value < 0.10 for the percent change from the mean of the measured outcome per unit IQR of exposure, **p value < 0.05 for the percent change from the mean of the measured outcome per unit IQR of exposure. (DOCX 21 kb) [file 12940_2017_335_MOESM2_ESM.docx]

|  | **Lag 0** | **Lag 1** | **Lag 2** | **Lag 3** | **Lag 4** | **5 day average** |
| --- | --- | --- | --- | --- | --- | --- |
| **LAEI** | | | | | | |
| PM_2.5_ | 2.5 (-4.8, 10.3) | 2.4 (-4.3, 9.7) | -2.3 (-8.2, 3.9) | -2.2 (-9.8, 6.0) | 1.8 (-5.1, 9.2) | 387 (-3.6, 11.7) |
| O_3_ | -1.9 (-16.8, 15.7) | -10.4 (-22.1, 2.9) | -8.8 (-19.6, 3.6) | 2.6 (-9.2, 15.9) | -11.3 (-21.8, 0.1)* | -21.1 (-35.6, -3.3)** |
| **SAEI** | | | | | | |
| PM_2.5_ | 1.1 (-8.8, 12.2) | -3.2 (-11.6, 6.0) | -4.1 (-11.6, 4.2) | -2.8 (-12.9, 8.4) | 3.4 (-6.2, 14.0) | -1.4 (-10.8, 9.1) |
| O_3_ | 13.0 (-10.2, 42.2) | 7.1 (-10.9, 28.7) | 18.1 (-1.4, 41.5)* | 15.9 (-1.8, 36.9)* | 3.4 (-13.2, 23.1) | 23.3 (-7.0, 63.5) |
| **FMD** | | | | | | |
| PM_2.5_ | -7.2 (-19.7, 7.3) | -0.4 (-13.4, 14.6) | 1.3 (-12.4, 17.1) | -7.4 (-22.9, 11.1) | -8.1 (-24.1, 11.1) | -6.3 (-20.8, 10.1) |
| O_3_ | -17.1 (-39.0, 12.8) | 4.4 (-26.1, 47.4) | -3.4 (-27.0, 27.9) | -19.5 (-39.3, 6.9) | 0.3 (-22.4, 29.5) | -17.2 (-49.2, 35.0) |
| **BAD** | | | | | | |
| PM_2.5_ | -0.1 (-1.6, 1.3) | 0.2 (-1.2, 1.7) | 0.0 (-1.2, 1.3) | 0.4 (-1.2, 2.0) | 0.7 (-0.7, 2.1) | -0.2 (-1.8, 1.5) |
| O_3_ | -2.1 (-5.2, 1.1) | -1.9 (-4.9, 1.2) | -2.5 (-5.1, 0.2)* | 1.7 (-0.7, 4.1) | 3.8 (1.4, 6.2)** | 1.8 (-3.0, 6.7) |
| **tPA** | | | | | | |
| PM_2.5_ | 0.3 (-3.4, 4.2) | 1.7 (-1.6, 5.1) | -1.6 (-4.5, 1.5) | -4.6 (-8.2, -0.9)** | -1.2 (-4.6, 2.3) | -1.6 (-5.3, 2.2) |
| O_3_ | 5.5 (-3.1, 14.8) | -1.2 (-7.5, 5.6) | 2.5 (-4.2, 9.7) | 6.7 (0.6, 13.1)** | 6.1 (-0.3, 12.9)* | 9.8 (-1.3, 22.2)* |
| **PAI-1** | | | | | | |
| PM_2.5_ | -5.7 (-14.5, 4.0) | 1.0 (-7.5, 10.4) | 4.2 (-3.7, 12.6) | 3.1 (-7.3, 14.7) | 7.3 (-2.0, 17.4) | 2.3 (-6.8, 12.4) |
| O_3_ | 7.8 (-13.1, 33.8) | 10.4 (-7.7, 32.0) | 20.6 (1.3, 43.6)** | 14.8 (-2.4, 35.0)* | 16.8 (-0.7, 37.4)* | 38.6 (6.5, 80.4)** |
| **# NEUTROPHILS** | | | | | | |
| PM_2.5_ | 0.6 (-3.3, 4.8) | 1.6 (-1.7, 5.0) | 2.0 (-1.2, 5.3) | 4.9 (0.8, 9.2)** | 2.9 (-0.6,6.6) | 2.8 (-1.0, 6.7) |
| O_3_ | 3.4 (-5.23, 12.9) | 8.4 (1.2, 16.1)** | 9.0n(1.6, 17.0)** | 4.0 (-2.2, 10.6) | -1.0 (-7.1, 5.5) | 9.5 (-1.9, 22.2) |
| **# MONOCYTES** | | | | | | |
| PM_2.5_ | -0.3 (-5.0, 4.7) | 1.8 (-2.3, 6.2) | 0.9 (-3.1, 5.1) | 2.3 (-2.8, 7.7) | 2.3 (-2.3, 6.9) | 1.8 (-2.9, 6.8) |
| O_3_ | 7.5 (-3.3, 19.5) | 9.8 (0.7, 19.8)** | 7.2 (-2.1, 17.2) | 0.3 (-7.3, 8.5) | -3.7 (-11.2, 4.4) | 8.4 (-5.8, 24.7) |
| **IL-6** | | | | | | |
| PM_2.5_ | 5.8 (-1.6, 13.8) | 2.2 (-3.9, 8.7) | 0.9 (-4.7, 7.0) | -0.9 (-8.0, 6.7) | -0.6 (-7.0, 6.2) | 3.6 (-3.6, 11.4) |
| O_3_ | 14.0 (-2.7, 33.5) | 6.6 (-6.1, 20.9) | 12.1 (-1.3, 27.2)* | 15.8 (3.5, 29.7)** | 0.1 (-11.1, 12.8) | 15.1 (-5.8, 40.8) |
| **TNF-α** | | | | | | |
| PM_2.5_ | 3.4 (-0.3, 7.4)* | 1.2 (-2.1, 4.5) | -0.2 (-3.1, 2.9) | 0.1 (-3.8, 4.2) | -0.8 (-4.1, 2.7) | 1.5 (-2.3, 5.3) |
| O_3_ | 6.5 (-1.8, 15.4) | 1.8 (-4.7, 8.8) | 5.9 (-0.9, 13.2)* | 1.4 (-4.6, 7.8) | -2.8 (-8.6, 3.4) | 3.4 (-6.8, 14.7) |

**Additional Table 2. Percent changes of measured factors with ambient ozone and PM_2.5_ concentrations using a two-pollutant model.** Effect estimates (95% CI) were log-transformed, correspond to changes per IQR of the corresponding pollutants, and were adjusted for season, temperature, and humidity. Effect estimates for FMD were also adjusted for the 5dMA barometric pressure. LAIE = large artery elasticity index; SAEI = small artery elasticity index; FMD = flow-mediated dilatation; BAD = baseline artery diameter; IL = interleukin; TNF = tumor necrosis factor; tPA = tissue plasminogen factor; PAI-1 = plasminogen activator inhibitor-1. *p value < 0.10 for the percent change from the mean of the measured outcome per unit IQR of exposure, **p value < 0.05 for the percent change from the mean of the measured outcome per unit IQR of exposure.
